# Sarcopenia, malnutrition, and fatigue in de Novo Parkinson’s disease– support for early rehabilitation

**DOI:** 10.1007/s10072-025-08317-1

**Published:** 2025-06-24

**Authors:** Beata Lindholm, Ylva Dernbrant, Albert Westergren, Erika Franzen

**Affiliations:** 1 Department of Neurology, Rehabilitation Medicine, Memory Disorders and Geriatrics, Skåne University Hospital Malmö, Malmö, 205 02 Sweden; 2 Cognitive Disorders Unit, Department of Clinical Sciences Malmo, Lund University, Malmo, 205 02 Sweden; 3 The PRO-CARE group, Faculty of Health Sciences, Kristianstad University, Kristianstad, Sweden; 4 Department for Research, Development and Education, Management for Psychiatry and Habilitation, Region Skåne, Lund, Sweden; 5 Division of Physiotherapy, Department of Neurobiology, Care Sciences and Society, Karolinska Institutet, Huddinge, Sweden; 6 Medical Unit Allied Health Professionals, Women’s Health and Allied Health Professionals Theme, Karolinska University Hospital, Stockholm, Sweden

**Keywords:** Early Parkinson’s disease, Indicators of sarcopenia, Malnutrition risk, Fatigue, Rehabilitation

## Abstract

**Objective:**

Sarcopenia (loss of muscle strength/mass) reduces physical performance (walking, strength, mobility) and is common in Parkinson’s disease (PD), but data on newly diagnosed, untreated patients (“de novo”) are scarce. Fatigue is frequent in PD and linked to physical decline and malnutrition, key sarcopenia risk factors. We aimed to describe sarcopenia prevalence, malnutrition risk, and fatigue in de novo PD and explore differences based on fatigue presence.

**Methods:**

Participants (*n* = 49) underwent physical tests [chair stand test (CST), hand grip strength, gait speed, Timed Up and Go test (TUG)] to assess sarcopenia. Malnutrition risk and fatigue were assessed using the Nutrition Form version 2 (MEONF-II) and the 16-item Parkinson’s Disease Fatigue Scale (PFS-16).

**Results:**

Twenty (40.8%) showed probable sarcopenia. Eight (16%) had fatigue, and 8 (19%) were at risk of malnutrition. No significant differences in sarcopenia indicators were found between groups, but those with fatigue had lower physical performance (CST, *p* = 0.033; TUG, *p* = 0.011), slower gait speed (*p* = 0.050), and poorer appetite (*p* = 0.003).

**Conclusion:**

A substantial proportion of de novo PD presented probable sarcopenia. Fatigue was associated with poorer physical performance and appetite but not sarcopenia. Whether fatigue increases risk of future malnutrition/sarcopenia is unclear due to cross-sectional study design. It should be further explored using a longitudinal study design.

## Introduction

Sarcopenia negatively affects physical performance and quality of life in older people. A gradual decline in skeletal muscle mass, strength, and endurance is a normal part of aging that can be exacerbated by malnutrition and lack of physical activity (PA) [1]. Early detection and intervention are crucial as they can help prevent the progression to overt sarcopenia. To this end, The European Working Group on Sarcopenia in Older People (EWGSOP2) has presented an updated consensus on defining and diagnosing sarcopenia. In these revised guidelines, it is acknowledged that muscle strength is a better predictor of adverse outcomes than muscle mass. Additionally, a practical algorithm with cut-off scores for physical tests relevant to clinical practice has been provided. Poor performance on the chair stand test (CST) or low hand grip strength (HGS) has been suggested as primary indicators of *probable* sarcopenia. In the next step, sarcopenia should be *confirmed* by evaluation of muscle quantity/quality and lastly walking at comfortable speed or Timed Up and Go (TUG) performance exceeding recommended cut-off scores indicates the *severity* of sarcopenia [1].

Parkinson’s disease (PD), an age-related progressive neurodegenerative disorder, arises from dysfunction of the basal ganglia, a group of subcortical structures whose primary function is the initiation and control of movements. Therefore, individual with PD must constantly manage worsening movement disorders such as tremors, stiffness, hypokinesia, and balance problems as well as a broad spectrum of non-motor symptoms. Due to the progressive nature of the disease physical performance in PD typically declines over time. Besides antiparkinsonian medication, PA is an essential component of PD care [2].

The prevalence of sarcopenia in PD appears to be associated with PD severity and is higher than in the general population [3]. It has been suggested that sarcopenia and PD share a common pathogenic mechanism [4]. However, the overlap between these two conditions may also be due to disease-related difficulties in maintaining normal levels of PA which is a common challenge even in the early stages of PD [5, 6]. Additionally, malnutrition and closely related fatigue [7], described as markedly diminished energy levels or a disproportionate feeling of effort during activities [8], may contribute to a sedentary lifestyle [9–11]. Since fatigue has been linked to physical decline [9] and malnutrition [7] in PD and is common even in the early stages of the disease [12] it could serve as a “red flag” for future declines in physical performance.

Although sarcopenia, malnutrition, and fatigue have been studied in PD [3, 13, 14] knowledge regarding newly diagnosed, untreated PD patients (“de novo”) remains scarce. As PD is the fastest-growing progressive neurological disorder globally [15], early identification of those at risk is of particular importance. Therefore, we aimed to describe sarcopenia prevalence, malnutrition risk, and fatigue in de novo PD and explore differences based on fatigue presence.

## Methods

### Participants

This was an observational study with a cross-sectional design. Seventy-four individuals with de novo PD receiving care at the outpatient neurology clinic at a university hospital in southern Sweden were identified and recruited by a movement disorder specialist between 2019 and 2022. PD was diagnosed according to the Movement Disorder Society (MDS) diagnostic criteria [16]. Eight individuals declined to participate. Seven participants were excluded due to severe comorbidity (*n* = 2) or an inability to understand instructions (*n* = 5). Ten participants were excluded due to missing fatigue data. The final study sample included 49 individuals with de novo PD. All included participants provided written informed consent. The study was approved by the Regional Ethical Review Board in Lund, Sweden (2018/412).

### Assessments and procedures

The included participants were invited to an outpatient clinic visit where they were assessed by a physiotherapist specializing in PD. Parkinsonian motor symptoms were evaluated using the Unified PD Rating Scale (UPDRS) part III (motor examination) which consists of 14 items graded from 0 to 4, with higher scores indicating greater symptom severity [17]. Disease severity was estimated using Hoehn and Yahr (H&Y) staging, which classifies PD progression into 5 stages, with stage 5 representing the most advanced stage [18]. Sarcopenia-related physical assessments, conducted according to EWGSOP2, included, the chair stand test (CST), handgrip strength (HGS), the 10-meter walk test (10MWT) at a comfortable speed, and the Timed Up and Go (TUG) test [1].

- CST, also known as the five times sit-to-stand test, measures the time required to stand up from a chair and sit down again five times. Participants were instructed to perform the test as quickly as possible with their arms crossed over their chest, while the time was recorded to the nearest 0.001 s using a digital stopwatch. The result serves as an indicator of skeletal muscle strength [19].
- HGS is another measure of skeletal muscle strength and was recorded was assessed using Grippit [20]. Both the left and right hands were tested twice, and the highest recorded value, measured in kilograms (kg) was used for analysis.
- The 10-meter walk test (10MWT) is a widely used assessment of gait speed, an indicator of physical function. In this study, participants were instructed to walk 10 m at a comfortable speed, starting from standing position [21]. Walking time was measured to the nearest 0.001 s using a digital stopwatch. Gait speed was then calculated in meters per second (m/s).
- The TUG test is a commonly used assessment of mobility. Participants were instructed to stand up from an armchair, walk three meters, turn around, walk back, and sit down [22]. The time taken to complete the test was recorded to the nearest 0.001 s using a digital stopwatch.

EWGSOP2 recommendations for sarcopenia cut-off scores are as follows: CST > 15 s, HGS < 16 kg for women and < 27 kg for men, gait speed ≤ 0.8 m/s, and TUG ≥ 20 s [1].

Additional data were collected using a self-administered questionnaire which included age, gender, as well as weight and height to estimate body mass index (BMI). Fatigue was assessed using the 16-item Parkinson’s Disease Fatigue Scale (PFS-16) [23] and nutritional status was evaluated using questions from the Minimal Eating Observation and Nutrition Form version 2 (MEONF-II) [24].

- PFS-16 is a scale developed specifically designed to detect fatigue in individuals with PD. It consists of 16 statements, each rated on a five-point Likert-scale: “strongly disagree”, “disagree”, “do not agree nor disagree”, “agree”, and “strongly agree”. Each response is scored from 1 to 5, with 1 representing “strongly disagree”, and 5 representing “strongly agree”. A PFS-16 score ≥ 2.95 indicates fatigue [23].
- MEONF-II is used to detect clinical undernutrition [25]. It includes questions yes/no questions covering three components of eating: ingestion, deglutition, and energy/appetite. Additionally, weight loss, BMI, and “clinical signs of undernutrition” (e.g. subcutaneous fat, muscle mass, grip strength) are assessed and scored as follows: 0–2 points (no or low risk), 3–4 points (moderate risk) and ≥ 5 points (high risk) [24]. Since MEONF-II was self-reported, we could not directly assess clinical signs. However, one point was assigned if participants exceeded the sarcopenia cut-off on at least one EWGSOP2 physical test, reflecting potential muscle function impairment. A MEONF-II total score of ≥ 3 was considered indicative of undernutrition risk.

### Statistical analyses

All analyses were made using IBM SPSS version 28. Continuous and discrete numerical variables were described using means and standard deviations (SDs) as well as medians (q1–q3). Categorical variables were presented as n (%). For between-group comparisons, the Mann–Whitney U test and chi-square test were used for numerical and categorical variables, respectively. The alpha level for statistical significance was set at 0.05 (two-tailed).

## Results

Of the participating patients, 42.9% (*n* = 21) were women, mean (SD; min-max) age was 67.1 years (10.80; 45–83). The corresponding values for UPDRS part III were 21.92 (9.68; 8–53) and the median (q_1_-q_3_) H&Y was 2.0 (2.0-2.5).

In total, 21 participants (42.9%) performed beyond cut-off scores for sarcopenia on at least one test. Twenty participants (40.8%) exceeded the sarcopenia cut-off score on the CST, five (10.2%) on gait speed, and two males on HGS. No participant exceeded the sarcopenia cut-off score on the TUG. Further details are presented in Table 1.

**Table 1:** De Novo Parkinson’s disease, *n* = 49. Indicators of sarcopenia and comparisons between those with and without fatigue^a^

	Whole group (*n* = 49)	Fatigue (*n* = 8)	No fatigue (*n* = 41)	*P*–value^b^
**Chair stand test**				
Mean (SD)	14.87 (5.16)	19.02 (5.89)	14.16 (4.75)^e^	**0.033** ^**c**^ *
Median (q_1_–q_3_)	14.28 (10.36–17.60)	18.14 (12.38–23.75)	13.85 (9.98–16.99)	
Above cut–off > 15 s, n (%)	20 (41.6)	5 (62.5)	15 (37.5)	0.111^d^
**Hand grip strength**				
Mean (SD)	34.16 (10.82)	28.75 (7.02)	35.22 (11.17)	0.121^c^
Median (q_1_–q_3_)	32 (26–40)	28 (22–37)	32 (26–42)	
Below cut–off < 16/<27 kg, n (%)	2 (4.1)	0 (0.0)	2 (4.9)	1.000^d^
**Gait speed**				
Mean (SD)	1.16 (0.25)	1.03 (0.15)^e^	1.19 (0.26)	**0.050** ^**c**^ *
Median (q_1_–q_3_)	1.16 (0.99–1.32)	1.04 (0.93–1.16)	1.21(1.01–1.30)	
Below cut–off ≤ 0.8 m/s, n (%)	5 (10.4)	1 (14.3)	4 (9.8)	1.000^d^
**Timed Up and Go**				
Mean (SD)	10.05 (2.51)	11.35 (1.92)^e^	9.44 (2.37)	**0.011** ^**c**^ *
Median (q_1_–q_3_)	9.62 (8.08–11.40)	10.83 (10.03–12.73)	8.92 (7.81–10.73)	
Above cut–off ≥ 20 s, n (%)	0 (0.0)	0 (0.0)	0 (0.0)	NA

^*^Significant at *p* ≤ 0.050

^a^ The 16–item Parkinson Fatigue Scale (PFS–16); cut–off > = 2.95 indicates fatigue

^c^Mann–Whitney U test

^d^Chi–square test

^e^One missing value

The mean (SD) PFS-16 score was 2.12 (0.96). Eight participants (16.3%) were classified as having fatigue (i.e. exceeding the cut-off score of ≥ 2.95). No significant differences between participants with and without fatigue in terms of age, gender, UPDRS III, or, H&Y stage (*p* ≥ 0.076).

There were no significant differences between groups regarding sarcopenia cut-off scores. However, the fatigue group required significantly more time to complete the CST (*p* = 0.033) and the TUG (*p* = 0.011) and had a significantly lower gait speed (*p* = 0.050). HGS did not differ significantly between groups.

Risk for undernutrition according to the MEONF II was identified in 8 individuals (18.6%). However, differences between participants with and without fatigue were not significant (*p* = 0.145). A significantly larger proportion of the fatigue group reported “problems with energy/appetite” (*p* = 0.003). There were no significant differences between groups for other instances in MEONF-II parameters or BMI. Further details are presented in Table 2.

**Table 2:** De Novo Parkinson’s disease, *n* = 49. Nutrition-related information and comparisons between those with and without fatigue^a^

	Whole group (*n* = 49)	Fatigue (*n* = 8)	No fatigue (*n* = 41)	*P*–value
BMI^b^				0.256^c^
Mean (SD) Median (q_1_–q_3_)	25.1 (3.8) 24.3 (22.3–27.6)	26.8 (4.9) 24.2 (22.2–27.0)	24.7 (3.6) 28.7 (21.1–30.1)	
**MEONF–II** ^**d**^				
Weight loss unintentional/unknown	9 (18.3)	2 (25.0)	7 (17.1)	0.639^e^
Low BMI^b^, n (%)	6 (12.2)	0 (0.0)	6 (14.6)	0.571^e^
Problems with intake, n (%)	6 (12.5)	1 (12.5)	2 (5.0)^f^	0.429^e^
Problems with swallowing, n (%)	0 (0.0)	0 (0.0)	0 (0.0)^g^	NA
Problems with energy/appetite, n (%)	3 (6.1)	3 (37.5)	0 (0.0)	0.003^e^ *
Risk of undernutrition, n (%)^5)^	8 (18.6)	3 (42.9)^f^	5 (13.9)^h^	0.145^e^

^*^Significant at *p* ≤ 0.050,

^a^The 16–item Parkinson Fatigue Scale (PFS–16); cut–off > = 2.95 indicates fatigue

^b^Body mass index (BMI); < 20 for < 70 years, or < 22 for ≥ 70 years is considered low

^c^Mann–Whitney U test

^d^The Minimal Eating and Nutrition Observation Form– version II (MEONF–II)

^e^Chi–square test

^f^One missing value

^g^Three missing values

^h^Five missing values

## Discussion

This study found that a significant proportion of individuals with de novo PD performed beyond the EWGSOP2 recommended cut-off scores for sarcopenia, indicating the occurrence of *probable* sarcopenia. Comparison between participants with and without fatigue did not reveal significant differences in sarcopenia indicators; however the fatigue group demonstrated poorer physical performance, and a greater proportion of individuals reported poor appetite.

In our de novo PD sample, the CST was the most prominent indicator of *probable* sarcopenia, with more than 40% performing beyond the recommended cut-off score. This result is largely in line with previously reported prevalence rates of 23.9–66.7% [3]. The discrepancy may be related to differences in PD severity among included individuals or methodical aspects. To our knowledge, this is the first study to evaluate sarcopenia in de novo PD using the EWGSOP2 cut-off scores.

According to EWGSOP2 guidelines, the CST can be used as a proxy for leg muscle strength. The repeated sit-to-stand movement (STS) in the CST challenges lower extremity strength and endurance [26, 27]. STS requires large hip and knee extensor muscle forces at the moment the buttocks lift off the chair [28, 29]. In fact, older adults have been reported to use up to 87-97% of their available knee strength to rise from a chair. Slow CST performance in PD has been associated with falls and low balance confidence, which may contribute to activity avoidance and a sedative lifestyle - a major risk factor for sarcopenia [1, 30]. Since muscle mass was not measured in this study, *probable* sarcopenia identified with CST could not be confirmed according to the EWGSOP2 algorithm. However, lower values of muscle mass, strength, and/or physical performance have been correlated with motor symptoms already in prodromal PD [4]. There is some evidence that the link between PD and sarcopenia could be driven by neuroinflammation mediated by interleukin-6 (IL-6). Therefore, normal age-related declines in muscle strength and mass may be more pronounced in PD, similar to what is observed in older adults with sarcopenia [31]. This underscores the importance of early monitoring of sarcopenia indicators in PD.

Some individuals with de novo PD in our study had gait speed below the recommended cut-off scores and could potentially meet the criteria for *severe* sarcopenia. Due to bradykinesia and hypokinesia, low gait speed is one of the cardinal motor impairments in PD. Initiating antiparkinsonian medication may improve gait speed and reduce motor symptoms; however, individuals with PD must still cope with reduced mobility and difficulties in maintaining normal levels of PA [5, 6]. Structured exercise has been shown to have positive effects on gait, balance, and muscle strength in PD [32, 33]. Although the evidence for sarcopenia treatment specifically in PD is yet to be established there is increasing consensus that sarcopenia should be managed using resistance exercises [3]. Thus, screening for indicators of sarcopenia in de novo PD may help guide patients toward early participation in neurorehabilitation programs.

The prevalence of fatigue in our sample was 16.3%, which is considerably lower than the estimated 50% in a previous meta-analysis that included individuals at all stages of PD [14]. However, different assessment tools have been used to evaluate fatigue, and our results are consistent with another recent study that also examined de novo PD using the PFS-16 [12].

Our findings suggest that individuals with de novo PD with fatigue did not perform worse on sarcopenia tests based on cut-off scores. However, results from the four physical tests for assessing muscle strength and physical performance allowed for a credible determination that participants with fatigue had poorer outcomes compared to those without fatigue. These findings suggest that the risk of sarcopenia may be present, at least among participants experiencing fatigue. Whether fatigue increases during the disease progress eventually leading more individuals to cross the cut-off thresholds should be evaluated using a longitudinal study design. In fact, studies have shown that individuals with PD and more severe fatigue are more sedentary and exhibit poorer physical performance compared to those with less fatigue [9]. The pathophysiology of fatigue in PD remains unclear. However, some evidence suggests that individuals with PD and fatigue have elevated levels of the neuroinflammatory mediator IL-6 compared to those without fatigue [34]. Sins IL-6 have been linked to both fatigue and sarcopenia in PD further investigation of a potential shared biological mechanism is warranted [31, 34]. This also underscores the importance of considering fatigue as a possible indicator of a more pronounced decline in muscle strength and mass in PD.

Additionally, a larger proportion of participants with fatigue had poor appetite compared to those without fatigue; however, the groups were similar regarding other nutritional risk factors, including weight loss, low BMI, swallowing difficulties and other problems with intake. BMI values suggest that individuals with PD and fatigue do not appear to be underweight at this early stage, despite their loss of appetite. This result aligns with a larger longitudinal Argentinian study that did not identify an association between decreased appetite and weight loss [35]. However, focusing solely on weight in individuals with PD is insufficient, as previous studies have noted that they might experience changes in body composition independent of weight change. For example, Vikdahl et al. [36] found an increase in fat mass over three years while skeletal muscle remained unchanged, leading to a relative loss of muscle mass. Similarly, Lindskov et al. [37] identified increased subcutaneous body fat and decreased muscle mass, while body weight remained the same over one year. A low muscle mass is, in itself, considered a sign of malnutrition [38]; therefore, the absence of weight loss could potentially mask the development of malnutrition. Since we did not measure body composition in this study, it remains unknown whether it differed between participants with and without fatigue, and whether poor appetite is related to body composition in PD is also unclear. However, a longitudinal study with a five-year follow-up found that loss of muscle strength exceeded the loss of muscle mass, possibly due to increased intramuscular fat [35]. Assessing muscle strength may therefore still be a useful tool in clinical practice for identifying the risk of malnutrition in PD. While our discussion includes evidence from previous studies on changes in body composition, we recognize that our study did not measure this directly and thus these considerations remain hypothetical.

Current evidence on nutritional interventions targeting PD and associated issues like sarcopenia remains inconclusive, primarily due to the scarcity of high-quality interventional studies. Although observational research and preliminary trials suggest potential benefits, such as improved management of non-motor symptoms and enhanced muscle health, the field is still in its early stages. Ongoing clinical trials are mostly exploratory or early-phase and focus on safety, biological responses, and symptomatic effects rather than definitive outcomes. Therefore, while nutritional strategies show promise, especially when considering their potential disease-modifying roles and impact on quality of life, robust randomized controlled trials are needed to establish clear recommendations and efficacy [39].

Since low PA and poor diet are known contributors to sarcopenia [1], it can be hypothesized that the poorer physical performance observed in the fatigue group could be attributed to fatigue affecting the dietary pattern, (e.g. a decreased energy or protein intake) and PA levels. However, the influence of fatigue on PA in PD remains uncertain. A recent Polish study did not find a correlation between fatigue and low PA levels [40], and another study investigating predictors of low PA found that fatigue was not an independent predictor [41]. Conversely, other studies have reported a significant correlation between fatigue and self-reported PA levels [9] as well as the number of daily steps [10] in PD. Elbers et al. [11] suggested that fatigue does influence PA levels but that the effect is very small. If so, this could explain the conflicting evidence. In summary, current evidence indicates the need of future studies exploring the efficacy of early rehabilitation programs, including resistance training and nutritional interventions, as well as evaluation of impact of fatigue to mitigate the risk of sarcopenia and malnutrition.

### Clinical implications

Since sarcopenia and malnutrition contribute to disease progression and disability, increasing the need for care, early interventions may improve the lives of individuals with PD and reduce healthcare costs. As the global prevalence of PD continues to rise rapidly [15], the disease will also become more common in clinical settings with strained resources and limited access to care. This underscores the importance of early identification of sarcopenia indicators and malnutrition risk to prevent these conditions from developing. Early screening for *probably* sarcopenia using the physical tests recommended by EWGSOP2 followed by evaluation of muscle quantity/quality, may be a feasible strategy to prevent adverse conditions.

### Limitations

The relatively small sample size, particularly the small number of individuals with fatigue, may limit the statistical power to detect group differences and reduces the generalizability of our findings. A larger sample size would also give possibility controlling for confounding variables such as age and sex. Additionally, it would be valuable to consider socioeconomic status and the presence of other medical conditions. Future studies should also incorporate objective measures such as blood tests or anthropometrics instead of self-administered MEONF-II that may give responses affected by recall bias or misinterpretation.

The exclusion of 10 participants due to incomplete fatigue data may have influenced the overall results and reduced the representativeness of our findings. Missing responses to some questions in the MEONF-II data may have introduced bias and affected the accuracy of our prevalence estimates.

Since our study did not include direct assessments of muscle mass or quality, we could only identify probable sarcopenia based on strength and physical performance measures, without confirming the diagnosis as per EWGSOP2 guidelines or fully exploring malnutrition aspects.

Finally, given the cross-sectional design, we cannot determine whether fatigue predicts sarcopenia or malnutrition over time. Our findings should be interpreted as associations rather than predictive relationships.

## Conclusion

A substantial proportion of individuals with de novo PD presented with *probable* sarcopenia. Fatigue occurred less frequently but was associated with poorer physical performance and appetite, which may reflect an increased risk of future sarcopenia and/or malnutrition, though longitudinal studies are required to confirm this. Also, intervention studies are warranted to evaluate the efficacy of targeted rehabilitation interventions to improve muscle strength, nutrition, and fatigue in patients with de novo PD.

## Data Availability

The data supporting the findings of this study are available on request from the corresponding author. The data are not publicly available due to privacy or ethical restrictions.
